# Case Report: Management of Multiple Deep-Tissue Cellulitis Without Sling Removal After an Anti-incontinence Procedure in a Female With Diabetes Mellitus

**DOI:** 10.3389/fsurg.2020.600754

**Published:** 2020-12-18

**Authors:** Hai-Hong Jiang, Sheng-Ping Hu, Yasmeen Bano, Ling-Xiao Ji, Peng-Fei Zhang, Kai Zhou

**Affiliations:** ^1^Department of Urology, The First Affiliated Hospital of Wenzhou Medical University, Wenzhou, China; ^2^Department of Radiology, The First Affiliated Hospital of Wenzhou Medical University, Wenzhou, China; ^3^Department of Gynecology, The First Affiliated Hospital of Wenzhou Medical University, Wenzhou, China

**Keywords:** tension-free vaginal tape transobturator (TVT-O), stress urinary incontinence (SUI), type 2 diabetes, cellulitis, synthetic sling

## Abstract

The transobturator suburethral tape procedure is emerging as a preferred surgical option in the management of stress urinary incontinence. This procedure, also called tension-free vaginal tape transobturator (TVT-O) procedure, has fewer risks of injury to the bladder, similar effectiveness, and shorter surgery duration compared with the older tension-free vaginal tape (TVT) procedure. In this study, we report the case of a female patient with type 2 diabetes mellitus who developed emergency ketoacidosis and severe cellulitis after a TVT-O procedure, which was successfully managed without sling removal and open drainage of abscesses after multi-point puncture drainage, guided by ultrasound and appropriate antibiotic administration. The patient showed appropriate urinary continence with controlled diabetes mellitus 24 months after treatment. In conclusion, cellulitis from the pelvic floor to the associated thigh after TVT-O procedure in a diabetic patient can be managed conservatively if no sling exposure is confirmed. However, these patients should be closely observed and followed up during the perioperative period, especially for synthetic sling use.

## Introduction

The tension-free vaginal tape transobturator (TVT-O) procedure is widely used for the surgical treatment of advanced stress urinary incontinence (SUI) in female patients because of minimal risk of urethral and bladder injuries (1). In this report, we describe the case of a patient with diabetes mellitus (DM) who developed pelvic floor and right limb cellulitis after TVT-O procedure. This report aims to share our experiences of providing treatment for emergency ketoacidosis and multiple deep-tissue cellulitis without an open incision and without removing the original synthetic sling.

### Case Presentation

**Figure 1:**
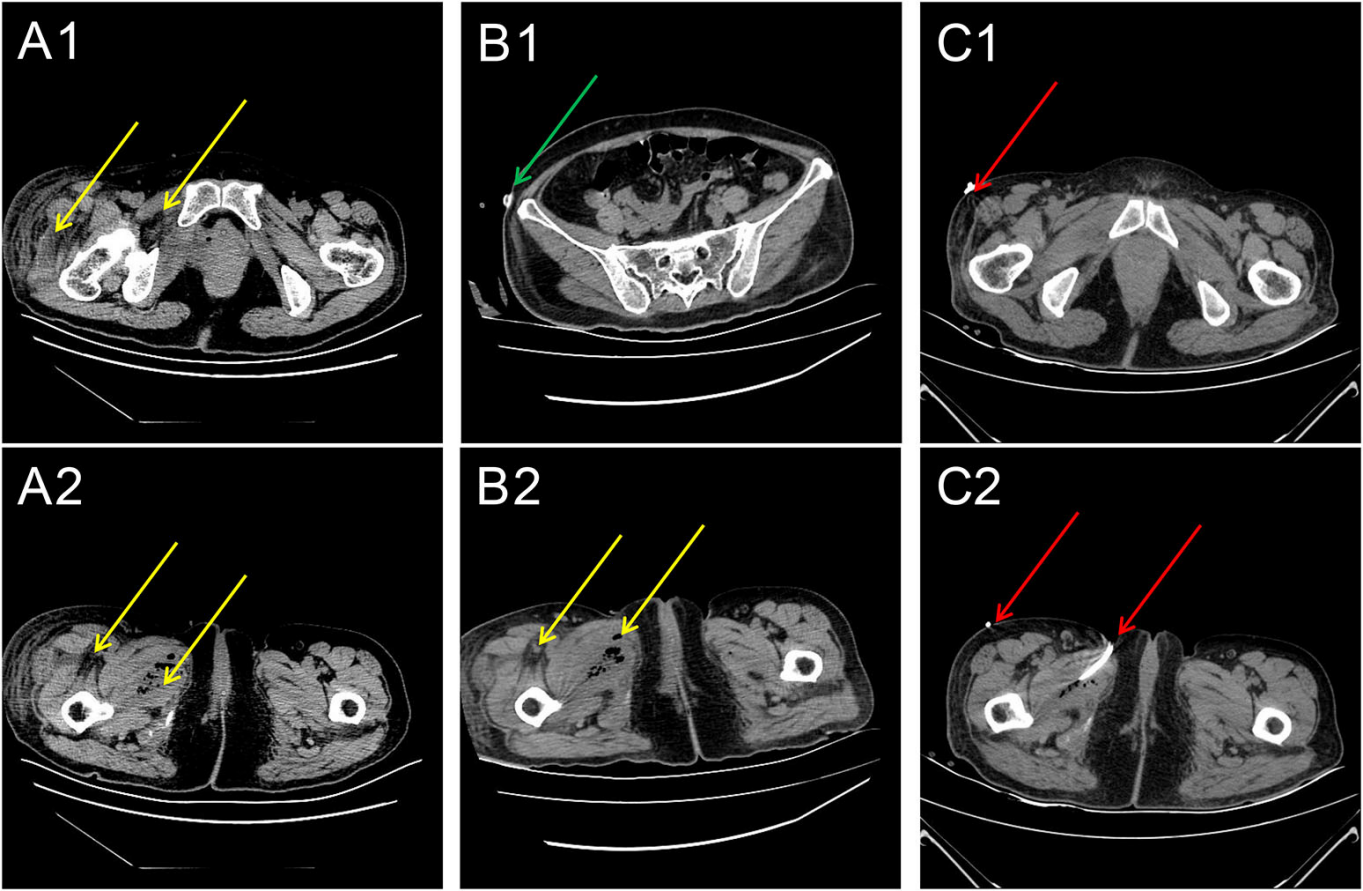
Series of computed tomography (CT) images **(A)** 1 week, **(B)** 2 weeks, and **(C)** 3 weeks after the sling procedure. **(A1,A2)** The CT scans of the pelvis and thigh regions indicate that the pelvic floor and thigh root muscle cavities have multiple gas shadows (yellow arrows). **(B1,B2)** The CT scans of the pelvis and thigh for assessing the infection status. The green arrows indicate the location of the drainage tube. The location of the gas (yellow arrow). **(C1,C2)** The CT scans of the pelvis and thigh for assessing the infection status. The red arrows indicate the location of the corrected drainage tubes. The location of the gas (yellow arrow).

**Figure 2:**
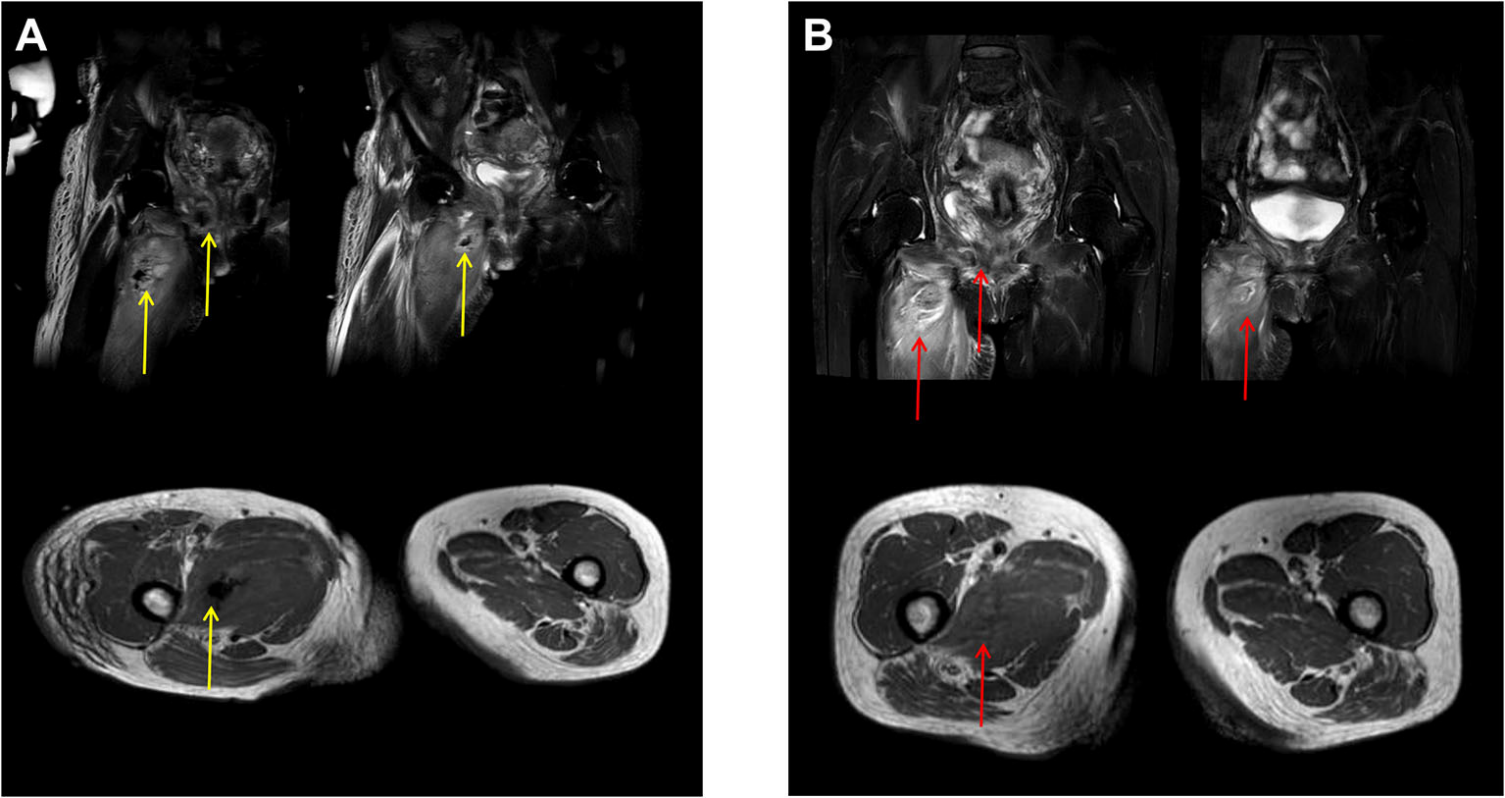
Magnetic resonance imaging (MRI) before and after tube drainage. **(A)** (Before drainage): (1) Infectious lesions observed in the right upper thigh skin and muscle group, right hip, right perineum, and pelvic floor muscle group accompanied by abscess in the right upper thigh muscle group and pelvic floor muscle group (gas shadow observed in the abscess); (2) Right lateral vaginal abscess; (3) The left lateral vaginal effusion shadow (yellow arrows). **(B)** (After drainage): no obvious abscess or gas observed in the upper part of the right thigh and the right pelvic floor muscle group (red arrows).

**Figure 3:**
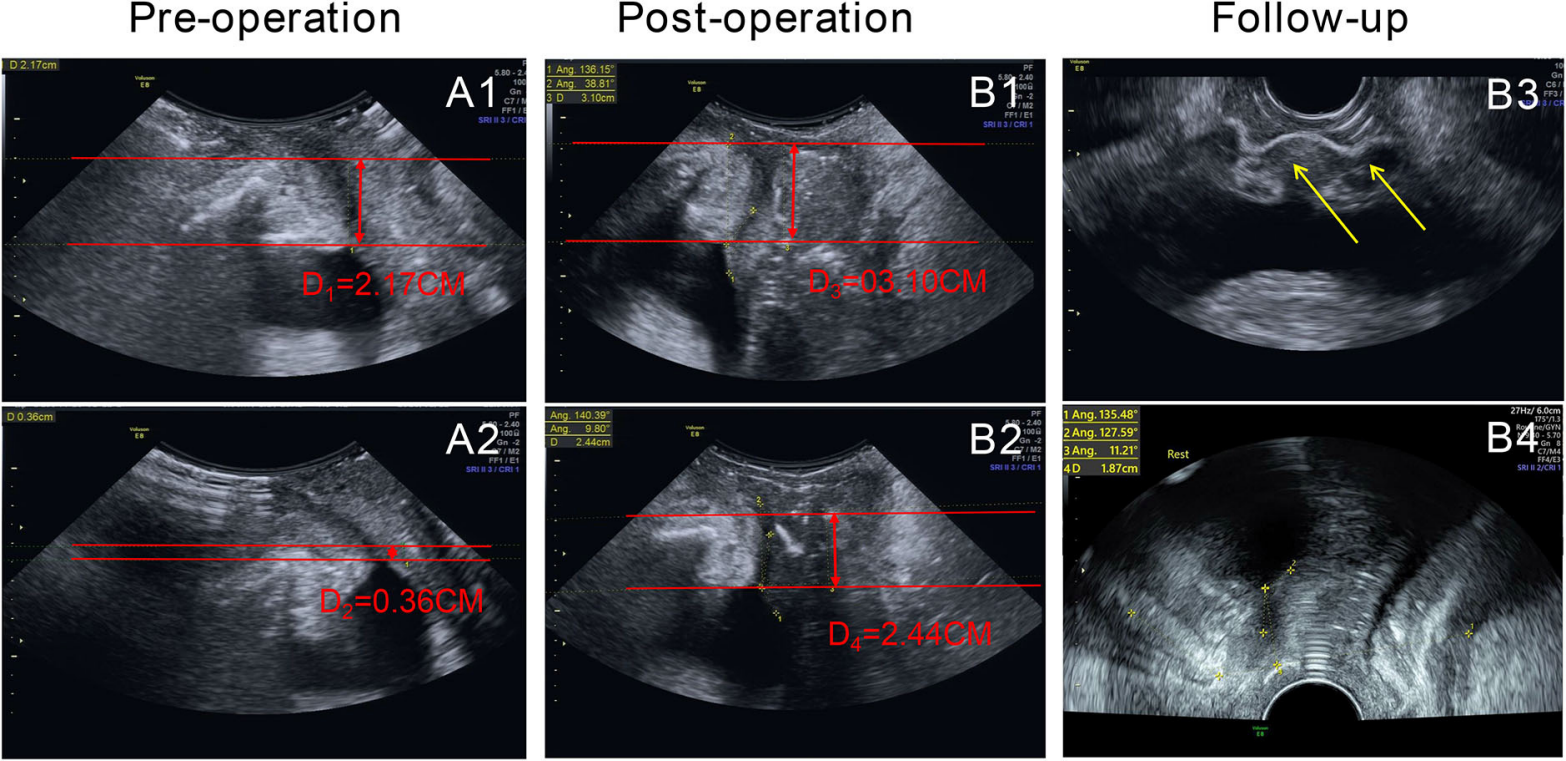
Results of ultrasonography before and after the procedure and drainage. **(A1,A2)** (pre-operation):1. Bladder neck funnel formation; the bladder neck drop distance is 18 mm (d_1_-d_2_, bladder neck is slightly prolapsed). **(B1–B4)** (post-operation and at follow-up): the bladder neck drop distance is 7 mm (d_3_-d_4_, no prolapse in the bladder neck). The completed sling is in the position at the middle sub-urethra (yellow arrows). There is no funnel formation in the bladder neck.

A 51-year-old woman with type 2 DM presented to the emergency department with dyspnea, dizziness, severe soreness in the right thigh, and difficulty walking. She had a temperature of 37.5°C on admission. She had been routinely discharged 1 week previously after a TVT-O procedure with synthetic sling implantation for the treatment of urinary incontinence. And this was a hospitalized patient. Antibiotics are used 0.5–1 h before routine surgery to prevent infection. The patient had the catheter removed on the second day after surgery. Before the procedure, the patient showed poor blood glucose monitoring records and an irregular medical treatment. After consultation at the department of endocrinology, insulin therapy was initiated, and her blood glucose spectrum was stable before procedure. The anti-SUI procedure was successful with no intraoperative or immediate postoperative complications. The patient was then discharged the following day after satisfactory urinary continence and voiding pattern. Her urine flow on uroflowmetry was 19.4 ml/s (Qmax) (voided urine: 204 ml; residual urine: 0 ml). However, she failed to comply with the requirements of blood glucose monitoring and glibenclamide medication. Upon admission, her right inner leg showed mild redness and swelling, especially close to the root of the thigh. Physical examination revealed extreme tenderness and stench of the right vaginal wall; however, the anterior vaginal wall incision remained intact with no obvious secretions. Diabetic ketoacidosis (DKA) and cellulitis (pelvic floor and right thigh) were diagnosed after a series of laboratory tests and imaging evaluations (Figure 1). Blood sugar analysis indicated blood glucose levels of 38.6 mmol/L, and her white blood cell count was 25.79 × 10^9^/L. An emergency computed tomography (CT) scan of the pelvis and thigh indicated multiple deep-tissue honeycomb-like gas shadow changes in the pelvic floor and bilateral thigh root muscle cavity (Figures 1, 2). Her right thigh was swollen but with no obvious crepitant rales. The circumference of the right thigh was 3 cm larger than that of the left thigh at the same level (Figure 3).

The abscess was drained by puncturing under ultrasound guidance, and laboratory examination of the drained fluid indicated infection with mixed anaerobic bacteria. Once the blood glucose levels, electrolyte levels, and acid-base balance of the patient were controlled, we initiated the administration of antimicrobial agents after consulting with the Infectious Disease Department for treating the infection. Furthermore, after multidisciplinary consultations, she was advised to continue active anti-infective therapy with multiple puncture point drainages under ultrasound guidance and close observation of cellulitis and DM conditions. With the stabilization of DM, the area of cellulitis was observed to have shrunk based on laboratory and imaging examinations. Her condition was gradually relieved with the use of antibiotics, glibenclamide, and supportive therapy. Magnetic resonance imaging (MRI) showed no collection of air bubbles in the thigh region or the pelvic floor. The patient showed complete recovery 4 weeks later and demonstrated maintained urinary continence and well-controlled DM on follow-up visits 3, 6, 12, and 24 months after discharge. Her DM has been well-controlled with medications. Further, she has shown no signs of infection, urine leakage, or any other related lower urinary tract symptoms. Her uroflowmetry results indicated a continuous bell-shaped urine flow with a detrusor thickness of 1.9 mm on ultrasound imaging. No residual urine was found in the bladder after urination on imaging. On ultrasound imaging of the pelvic floor, a corrected bladder neck funnel (3 mm above the reference line) was observed with a continuous shadow of the sling under the mid-urethral area, ~16 mm away from the internal orifice (Figure 3).

## Discussion

In this study, we report the case of an adult woman with DM who developed cellulitis because of poor diabetes management after TVT-O procedure. During management, neither removal of the sling, which was a foreign body, nor extensive incision or drainage was performed, and the condition was mainly resolved with close surveillance. No sling exposure or erosion was observed, and the patient maintained well-functioning urinary continence and voiding.

The TVT-O procedure has become a standard solution for the surgical treatment of severe SUI among adult women (2). Although this procedure has a relatively low risk of injury and postoperative complications, anti-incontinence procedures, such as TVT-O, may result in a variety of complications (3). Further, soft tissue-related infections in the thigh are very uncommon, and usually indicate a severe complication (4). Goldman, in 2005, described a similar case, wherein a 44-year-old woman developed cellulitis on the inner thigh and fever on postoperative day 1. Despite a course of antibiotics, an abscess formed in the adductor muscles, and pus was seen draining from the vagina. The sling was removed with the belief that the vagina was likely the source of the infection (5).

A vaginal surgery is considered to be a “clean-contaminated surgery” because the vagina is naturally colonized with bacteria. Normal flora comprise various microorganisms such as *Lactobacillus coryniformis*, anaerobic bacteria, *Staphylococcus* spp., *Streptococcus* spp., *Enterococcus faecalis, Escherichia coli*, and *Klebsiella pneumoniae* (6). In our case, vaginal pathogens may have escaped through the well-sutured incision and spread rapidly because of the underlying uncontrolled DM and surgical stress. The affected areas, based on the imaging results, included the mid and upper parts of the right thigh, right hip, right perineum, and left hip, which indicated the area around the sling-related compartments. It is interesting to note that the areas most affected by infection were located on the right side. We believe that the compartments on the pelvic floor are rigid and limited; thus, the infection spread to the thigh on the side where the obturator foramen was punctured. This indicates that such an infection usually would not spread beyond the pelvic floor or peritoneal compartment, even when typical emphysematous cystitis with a fuzzy bladder wall is observed under imaging. Therefore, under our intensive care, we observed that the infection within the compartment eventually resolved with appropriate treatment. This may indicate that such complications can be managed without sling removal, even in rare and life-threatening conditions complicated with diabetic ketoacidosis (DKA) in a patient with type 2 DM. However, this case helps us understand that DM with no appropriate blood glucose control after surgical sling implantation may put the patient in a very dangerous situation. CT or MRI of the pelvis and thigh root can be useful for diagnosing the extent of the infectious process, and a series of examinations may help monitor whether surgical removal of the tape is needed (4).

In our practice, treatment for surgical anaerobic infections should focus on controlling the foci of infection, removing inactivated tissue, and draining the pus and gas (7). This will lead to the anaerobic bacteria losing their growth conditions under oxygen tension (8). Conservative management has been recommended in cases of small vaginal erosions and surgical management in cases where there is no improvement with conservative approaches (9). In our patient, the vaginal incision was well-sutured with corrected urinary incontinence and no sling exposure. Therefore, instead of removing the sling, we closely observed the patient and provided active anti-infective therapy. The patient recovered completely without the requirement of opening the incision or removing the sling. Thus, we conclude that timely and effective control of infections is still the preferred choice over surgical treatment for anaerobic infections in most situations (10). However, it should be highlighted that such complications can be handled without explanation only in cases without fistulas or injuries to the pelvic structures. Further, some other limitations of this study arise from the nature of the study, which is a Case Report. These include the lack of the ability to generalize the results, no possibility of establishing a cause-effect relationship, possibility of over-interpretation and publication bias. Accordingly, the approach reported in this study needs to be continuously verified in practice in the future.

Based on this case management, we believe that blood glucose control is a key to appropriate perioperative treatment, especially for avoiding complications such as infections, which could induce life-threatening DKA and cellulitis. Instead of removing the sling, opening incision drainage and adequate puncture drainage with vacuum suction combined treatments for DM and infection can lead to complete recovery of patients as long as they are closely monitored. Nevertheless, we also agree that surgical intervention should be initiated if vaginal erosion or sling exposure is presented with severe uncontrolled infection.

## Data Availability Statement

The original contributions presented in the study are included in the article/supplementary material, further inquiries can be directed to the corresponding author.

## Ethics Statement

Written informed consent was obtained from the individual(s) for the publication of any potentially identifiable images or data included in this article.

## Author Contributions

H-HJ performed the surgery, managed the patient, and wrote the manuscript. KZ edited the manuscript and reviewed the patient management. YB, S-PH, P-FZ, and L-XJ collected the data and images and edited the article. All authors contributed to the article and approved the submitted version.

## Conflict of Interest

The authors declare that the research was conducted in the absence of any commercial or financial relationships that could be construed as a potential conflict of interest.
